# Comparing Surrogates to Evaluate Precisely Timed Higher-Order Spike Correlations

**DOI:** 10.1523/ENEURO.0505-21.2022

**Published:** 2022-06-08

**Authors:** Alessandra Stella, Peter Bouss, Günther Palm, Sonja Grün

**Affiliations:** 1 Institute of Neuroscience and Medicine (INM-6) and Institute for Advanced Simulation (IAS-6) and JARA Institute Brain Structure-Function Relationships (INM-10), Jülich Research Centre, 52428 Jülich, Germany; 2 Institute of Neural Information Processing, Ulm University, 89069 Ulm, Germany; 3 Theoretical Systems Neurobiology, RWTH Aachen University, 52062 Aachen, Germany

**Keywords:** massively parallel spike recordings, neural code, significance evaluation, spatiotemporal spike patterns, stochastic point processes

## Abstract

The generation of surrogate data, i.e., the modification of data to destroy a certain feature, can be considered as the implementation of a null-hypothesis whenever an analytical approach is not feasible. Thus, surrogate data generation has been extensively used to assess the significance of spike correlations in parallel spike trains. In this context, one of the main challenges is to properly construct the desired null-hypothesis distribution and to avoid altering the single spike train statistics. A classical surrogate technique is uniform dithering (UD), which displaces spikes locally and uniformly distributed, to destroy temporal properties on a fine timescale while keeping them on a coarser one. Here, we compare UD against five similar surrogate techniques in the context of the detection of significant spatiotemporal spike patterns. We evaluate the surrogates for their performance, first on spike trains based on point process models with constant firing rate, and second on modeled nonstationary artificial data to assess the potential detection of false positive (FP) patterns in a more complex and realistic setting. We determine which statistical features of the spike trains are modified and to which extent. Moreover, we find that UD fails as an appropriate surrogate because it leads to a loss of spikes in the context of binning and clipping, and thus to a large number of FP patterns. The other surrogates achieve a better performance in detecting precisely timed higher-order correlations. Based on these insights, we analyze experimental data from the pre-/motor cortex of macaque monkeys during a reaching-and-grasping task.

## Significance Statement

Temporal jittering or dithering of single spikes or subsections of spike trains is a common method of generating surrogate data for the statistical analysis of temporal spike correlations. We discovered a serious problem with the classical and widely used method of uniform dithering (UD) that can lead to an overestimation of significance, i.e., to false positives (FPs) in the statistical evaluation of spatiotemporal spike patterns. Therefore, we consider five other dithering methods, compare and evaluate their statistical properties and test them on increasingly complex data. Finally, using the most robust surrogate method (trial shifting; TR-SHIFT) in the analysis of experimental multiple-unit recordings, we find several highly significant patterns reflecting different experimental contexts.

## Introduction

The usage of surrogates has become a standard tool in data analysis and computational statistics (Abeles and Gat, 2001; Grün et al., 2002a; Grün, 2009; Louis et al., 2010b). It is often used to replace the definition of an appropriate null-hypothesis in classical statistical testing, which describes the possibility that the observed effects or results have occurred merely by chance. In statistical textbooks and software, standard definitions of null-hypotheses are used, which allow the analytical derivation and automatic computation of the corresponding significance probabilities. In computational statistics, it has become possible to determine these probabilities also for more complex and not analytically tractable null-hypotheses, by extensive sampling from the null-hypothesis distribution, i.e., by generating artificial data from this distribution. The generation of surrogate data follows a completely different approach. In exploratory data analysis or scientific investigations, we may have observed an interesting effect, but we often have no idea what would be an appropriate model for “randomness,” and it would be premature to assume a standard random model, like the normal distribution, just for convenience. In this situation, we can use the data themselves to test for the significance of the observed effect by simply modifying them to generate more data of the same type, which can then be used to determine significance probabilities. Such methods are called bootstrapping (Efron and Tibshirani, 1993), and typical methods consist in resampling or reordering the data, or adding small amplitude noise. The generation of surrogate data is a particular version of this, which is typically used when we have an idea or hypothesis concerning the features in the data that are relevant for the effect. In this case, we modify or add some noise to the data to destroy these features. If we use these modified data as our “null-hypothesis” and the observed effect does not occur or occurs with a very low probability, we have obtained evidence that those features are indeed relevant and our hypothesis was correct (Kass et al., 2005; Ventura, 2010).

Here, we are interested in the interactions between hundred or more neurons that were recorded in parallel by multiple electrodes (Riehle et al., 2013; Brochier et al., 2018). In view of the apparent randomness in the reaction of neurons to repeated stimuli (Nawrot et al., 2008), one important question concerns the temporal precision of neural interactions, which has been studied by means of pairwise (Grün et al., 2002a,b; Pipa and Grün, 2003; Pipa et al., 2003, 2007, 2013; Grün, 2009) and higher-order correlation analysis (Villa and Abeles, 1990; Martignon et al., 1995; Riehle et al., 1997; Prut et al., 1998; Villa et al., 1999; Kilavik et al., 2009; Shimazaki et al., 2012) of parallel recorded spike trains. In order to demonstrate high precision in temporal multiple-neuron interactions by statistical methods, one needs surrogate methods that destroy correlations at high temporal precision but not at low temporal precision, and that maintain as much as possible all other statistical properties of the individual spike trains. Thus, the basic idea was to slightly perturb the spike times of the data. These surrogate methods are called dithering or jittering or teetering (Date et al., 1998; Hatsopoulos et al., 2003; Pazienti et al., 2008; Stark and Abeles, 2009; Louis et al., 2010b); the most commonly used of these methods is uniform dithering (UD), which shifts each individual spike by a small uniformly distributed amount. Unfortunately, this method suffers from severe problems concerning the preservation of statistical properties like the interspike interval (ISI) distribution and can lead to substantial spike count reduction and therefore to an underestimation of pattern significance probabilities when combined with binarization of spike trains, which is a prerequisite for many methods of statistical analysis. Consequently, we introduced some different surrogate methods and compared them with each other and with UD in terms of statistical properties, and in particular the effect on significance evaluation of repeating spatiotemporal spike patterns Spike PAttern Detection and Evaluation, or SPADE; Torre et al., 2013; Quaglio et al., 2017; Stella et al., 2019].

For this comparison, we use artificially generated data of increasing complexity where repeated spatiotemporal patterns can only occur by chance so that we can observe the amount of false positive (FP) patterns. Finally, we analyze the experimental data for “true positives” using the six different surrogates.

## Materials and Methods

### Surrogate methods

Generally, surrogate data are used as an implementation of a null hypothesis in statistical analysis when there is no analytical or generative probabilistic model available. Dithering methods, in particular, are used to differentiate between correlations across multiple spike train data that contain modulated firing rates (on a timescale of tens of milliseconds) and those based on fine temporal spike correlation (at a timescale of milliseconds). For example, when single spikes are shifted by <25 ms, independently of each other, one can assume that all dependencies on the millisecond scale are destroyed, whereas all dependencies on a coarser time-scale, i.e., the time-scale of firing rate estimation, are kept. This includes all correlation structures between different neurons, but also between neurons and external stimuli or conditions on a time resolution of 25 ms or coarser.

Per default, we make sure that the considered surrogates preserve the firing rate modulations of the original data; in addition, we put our emphasis on preserving also other features of the spike trains, such as Inter-Spike Intervals (ISIs) and derived measures, since it is not yet clear what effect a disturbance of ISIs may have on pattern significance. In principle, one of course wants to keep all the features of the spike trains, besides their correlation, but this is not possible. Thus, we aim to test here which of the dithering manipulations are leading to the least FPs.

Different types of surrogates were already developed, however, in the context of different analysis methods (Gerstein, 2004; Pipa et al., 2008, 2013; Grün, 2009; Louis et al., 2010a,b). Here, we compare six different surrogate methods, four known from the literature and two newly developed by us, and evaluate their applicability for significance assessment in spike train correlation analyses. We do not consider surrogates that destroy the firing rate profile, since we give particular attention to surrogate techniques that are supposed to preserve as many features of the individual spike train as possible, e.g., the ISI distribution and parameters derived from it such as the coefficient of variation (CV).

#### Uniform Dithering

**Figure 1.**
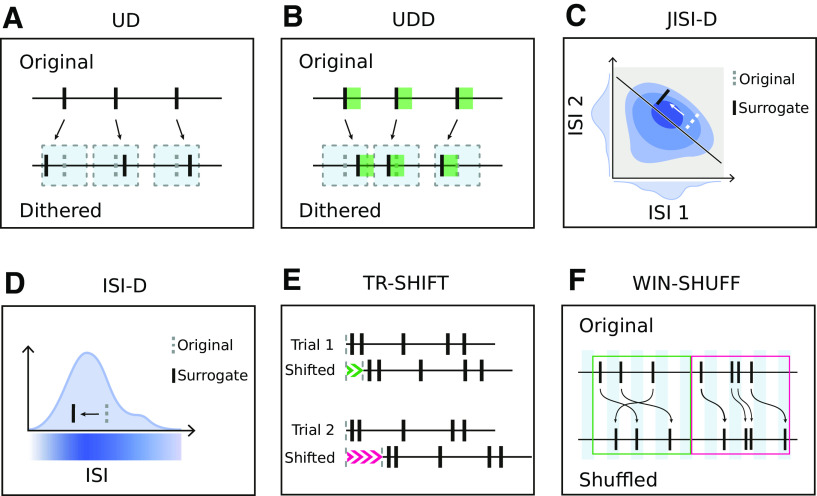
Sketches of the surrogate techniques. ***A***, UD. Each spike is displaced according to a uniform distribution centered on the spike. Gray dotted rectangles represent the dithering window. ***B***, UDD is similar to UD, but spikes are constrained not to be closer to each other than a given dead-time. Green shadows represent the dead-time after each spike. Gray dotted rectangles represent the dithering window. ***C***, JISI-D. Each spike is displaced according to the JISI distribution of the neuron, sampled from the data. JISI distribution, projected in a two-dimensional plane, is represented in blue. On the *x-* and *y*-axes, we represent the projections of the first and second ISI given three spikes. ***D***, ISI-D. Each spike is displaced according to the ISI distribution of the neuron, sampled from the data. The ISI distribution is represented in blue, along with its intensity. ***E***, TR-SHIFT. Each trial is shifted by a randomly chosen amount from a uniform distribution (represented in green and pink), independently across trials and neurons. ***F***, WIN-SHUFF. Binned spike data are shuffled within exclusive windows (marked green and pink).

The UD method consists in displacing each individual spike of each neuron by a small uniformly distributed random jitter ∼*U*[– Δ, +Δ] around its original position. An example sketch is shown in Figure 1*A*. It is also known by the names jittering or teetering and is a classical choice for surrogate generation and was employed in several experimental studies (Abeles and Gat, 2001; Hatsopoulos et al., 2003; Gerstein, 2004; Shmiel et al., 2006; Maldonado et al., 2008; Torre et al., 2016a,b). Because of its simplicity and computational speed, it was widely used for detection of pairwise synchrony (i.e., cross-correlogram significance estimation; Grün, 2009; Louis et al., 2010b), higher-order synchrony, and pattern detection (Abeles and Gat, 2001; Gansel and Singer, 2012; Torre et al., 2016a,b). In particular, it was chosen as the surrogate generation technique for synchrony and pattern detection using SPADE (Torre et al., 2016a; Quaglio et al., 2017; Stella et al., 2019). However, Louis et al. (2010a) already demonstrated in the context of pairwise spike synchrony analysis that UD can lead to FPs for regular firing properties (CV < 1).

The dither parameter of the method Δ > 0 determines the maximal displacement of a spike from its original position. It needs to be selected appropriately, e.g., in the range of 15–25 ms (Torre et al., 2016a; Stella et al., 2019) and is typically a multiple of the bin size parameter. If Δ is too small, it causes insufficient displacement of the correlated spikes and may lead to an underestimation of significance, whereas if Δ is too large, it yields a strongly smoothed firing rate profile (Pazienti et al., 2008) and, thus, an inappropriate null-hypothesis (Louis et al., 2010b).

#### Uniform Dithering with dead-time (UDD)

We introduce UDD as a variant of the classical UD (Fig. 1*B*), because we noticed that experimental data often have a dead-time after a spike of up to 2 ms, and we wanted to keep this property in the surrogate data. Thus, we estimate the dead-time *d* from the experimental data and conserve it during the temporal displacement of each spike. This is done by limiting the window into which spikes can be dithered. The uniform displacement of each spike is limited by the intervals to the neighboring spikes minus the dead-time *d*. Thus, it does not allow two dithered spikes to have a temporal distance smaller than the dead-time, and, unlike for UD, the displacement of each spike is hence not independent of its neighbor. As described later (in Results, Origin of spike count reduction), a dead-time may be introduced by spike sorting. Further, the biological absolute refractory period of neurons can yield minimal intervals larger than those inserted by the spike sorting. We estimate the dead-time for each neuron to be the minimum ISI across all trials. In case of low firing rates, the minimum ISI may be still in the range of hundreds of milliseconds, complicating the estimation of a biologically reasonable dead-time. For this reason, we define a maximal dead-time parameter *d_max_* such that, if the minimal ISI exceeds *d_max_* (in our case 4 ms), then we set *d* = *d_max_*.

#### Joint-ISI dithering (JISI-D)

Gerstein (2004) suggested to dither spikes of adjacent ISI intervals to keep the distribution of the preceding and following ISIs relative to a spike according to the joint-ISI probability distribution (JISI-D; Fig. 1*C*; Gerstein, 2004; Louis et al., 2010a). This probability distribution is derived from the original data for each neuron by calculating the corresponding JISI histogram (with a default bin size of 1 ms). Dithering one spike according to such a two-dimensional histogram corresponds to moving the spike along the anti-diagonal of the JISI distribution (Gerstein, 2004; Louis et al., 2010a).

Unfortunately, experimental recordings are often nonstationary and too short to comprise enough spikes to estimate the underlying JISI probability distribution. Therefore, we apply on the JISI histogram a 2d-Gaussian smoothing with variance *σ*^2^, with *σ* of the order of milliseconds (Louis et al., 2010a).

#### ISI dithering (ISI-D)

ISI-D (Fig. 1*D*), unlike JISI-D, does not consider the pair of a current and its subsequent ISI, instead, it dithers the individual spikes according to the ISI probability distribution and ignores its sequence. However, for practical reasons, we implemented ISI-D as a special case of the JISI-D assuming that two consecutive ISIs are independent, i.e., that the JISI histogram can be represented as the product of the ISI histogram with itself $p_{\text{JISI}}(\tau ,\tau^{\prime })=p_{\text{ISI}}(\tau )\cdot p_{\text{ISI}}(\tau^{\prime })$. Thus, in comparison to the JISI-D, ISI-D does not take into account the correlations of subsequent ISI pairs and is particularly useful when there are not enough data to estimate the JISI distribution. As a result, not the distribution of pairs of subsequent ISIs are preserved, but the distribution of the single ISIs regardless of their order.

#### Trial shifting (TR-SHIFT)

As an alternative to the dithering of single spikes, Pipa et al. (2008) introduced dithering of the entire spike train. This has the advantage that firing rate and ISI structure in the data are completely kept intact, but potential correlations across spike trains are destroyed. TR-SHIFT (Fig. 1*E*; Pipa et al., 2008; Louis et al., 2010b) consists of shifting all spike times identically by a random uniform amount ∼*U*[– Δ, +Δ], independently neuron by neuron and trial by trial. The method requires the time randomization to be different across neurons in the different trials, such that repeating identical patterns are shifted into different patterns from trial to trial. Therefore, the method requires a segmentation of the spike trains into longer temporal sequences, called trials here. These could also be longer spike sequences that are separated by relatively long intervals between spikes as suggested by Harrison and Geman (2009). In our case, the trials are defined by the experimental protocol. TR-SHIFT has the benefit of keeping the entire spike train structure intact during each trial.

#### Window shuffling (WIN-SHUFF)

Finally, we designed a method that, by construction, preserves the spike count of the discretized original spike train in all surrogate realizations. We introduce WIN-SHUFF (Fig. 1*F*), which divides the spike train into successive and exclusive small windows of predefined duration Δ _WS,_ and further divides the windows into bins of length *b* (Δ_WS_ should then be a multiple of *b*). The bins are then shuffled within each window. Additionally, spike times are randomized within each bin. The firing rate profile is modified by the local shuffling of the spikes to be stationary inside in window of duration Δ_WS._ To facilitate the comparison to the other methods, we fix throughout the paper Δ_WS_ = 2Δ.

### SPADE

One important application of the surrogate methods compared here is a software for the evaluation of spatiotemporal spike patterns that occur repeatedly in multiple unit recordings. This software, called SPADE (Torre et al., 2013; Quaglio et al., 2017; Stella et al., 2019), is quite complex and described here. It combines the extensive use of dithering techniques with a computer science method [frequent itemset mining (FIM); Borgelt, 2012]. SPADE has to resort to this rather technical method because there is usually a huge number of repeating patterns that will be found also for each of the surrogate datasets.

**Figure 2.**
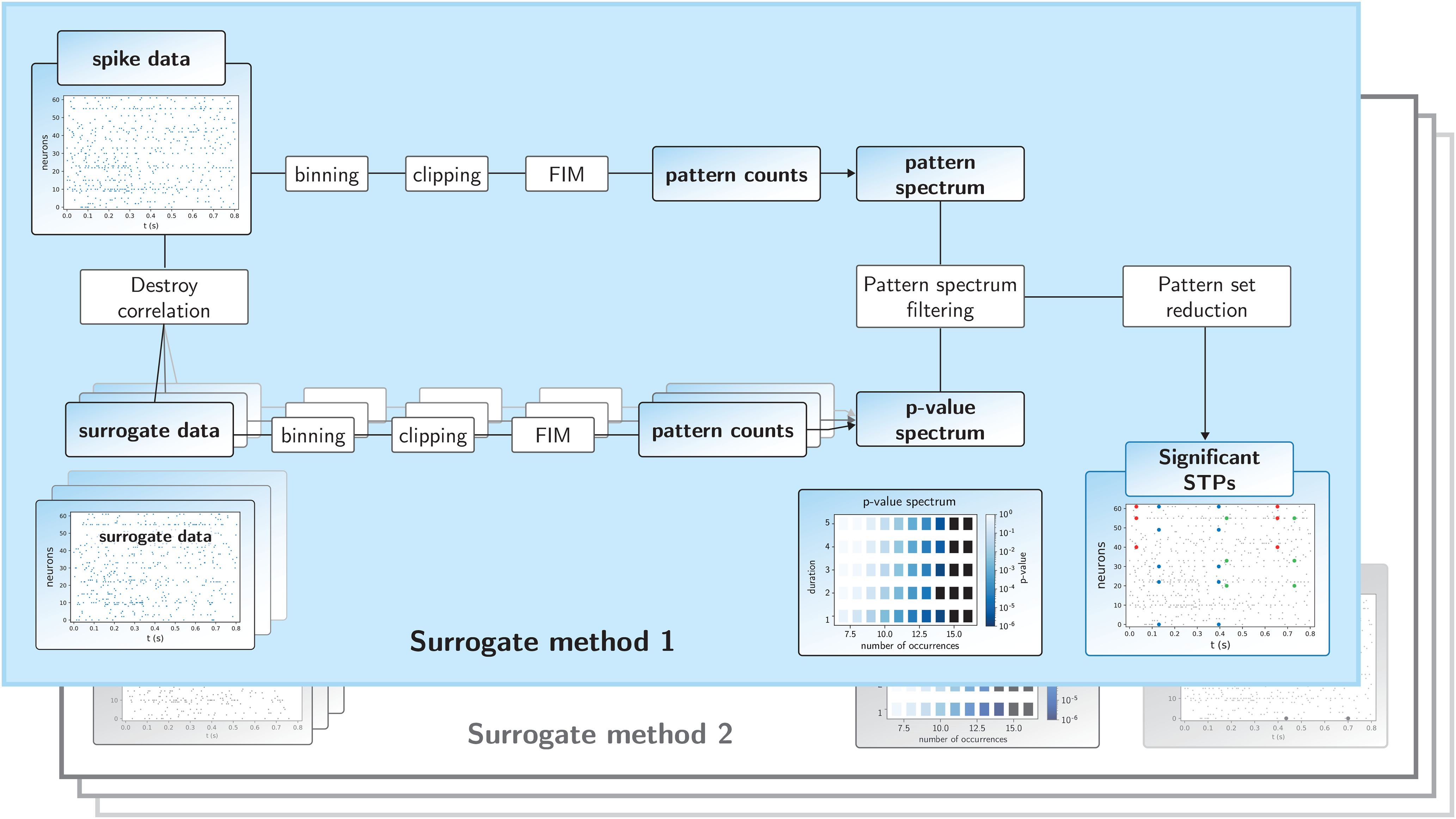
Workflow of the SPADE analysis. The top branch of the SPADE workflow shows the sequence of analysis steps of the original data until the pattern spectrum is derived. The bottom branch of the workflow starts with the generation of the surrogate data from the original data, followed by the same analysis steps as for the original data. The multiple overlapping panels in the lower branch indicate that this surrogate procedure is repeated many times, by which the *p*-value spectrum is built up. This then serves for the extraction of significant patterns through PSF. After the application of the pattern set reduction, significant STPs are provided as a result. “Surrogate method 2” indicates that the part “Destroy correlation” and “surrogate data” are replaced by another surrogate method, but the other steps stay the same.

The spike train data are first discretized into exclusive time intervals (bins). Typically, the bin length consists of a few milliseconds, which at the same time defines the allowed temporal imprecision of neuronal coordination. The procedure of discretization counts the number of spikes within each bin (binning, Grün et al., 2002a; Torre et al., 2013), followed by reducing the bin content to 1 if a bin contains >1 spike (clipping). In the following, we will call the combination of these two steps binarization. Candidate spatiotemporal patterns are then mined using the FIM algorithm (Zaki, 2004; Borgelt, 2012; Picado-Muiño et al., 2013), which yields the number of occurrences of each nontrivial detected spike pattern, along with its occurrence times. A nontrivial pattern is defined as one that, repeats at least a fixed number of times but cannot be explained as part of a larger pattern. Pattern counts are then collected in the so-called pattern spectrum, i.e., the pattern counts are entered in a 3d-histogram according to their number of spikes *z*, the number of pattern repetitions *c*, and the temporal extent from first to last spike *d* (Fig. 2). The triplet (*z*, *c*, *d*) is called the signature of the pattern. Thus, for example, a pattern composed of four spikes of four different neurons, occurring 29 times, with a duration of 10 bins (50 ms) has a signature of *z *=* *4, *c *=* *29, *d *=* *10. Other patterns with identical signature are counted in one entry. Thus, each pattern enters in a particular entry of the 3d pattern spectrum.

This pooling of the patterns found by FIM according to their signature is a crucial step for significance testing because it avoids testing each pattern individually, but rather considers the probability of generating a pattern with a given signature. This reduces enormously the number of tests to be considered in the multiple testing correction.

FIM efficiently collects and counts pattern candidates, nonetheless, the statistical significance of each of the mined patterns has still to be evaluated. SPADE aims at testing whether the patterns emerge as an effect of precisely timed neuronal coordination, or merely by random spike generation governed by the firing rates. In this sense, the null-hypothesis is that spike trains are mutually independent given their firing rate (co-)modulations and that the occurring patterns in the data are given by chance.

For the significance evaluation of the patterns found in the original data (Fig. 2, upper branch), we make use of surrogate data (Fig. 2, lower branch of the workflow). A surrogate is generated from the original data by, e.g., UD. Then, surrogate data are analyzed in the same way as the original data (binning, clipping, FIM, pattern counts), and the counts are entered in the pattern spectrum. After, e.g., 5000 surrogates, a 3d-*p*-value spectrum is computed by binarizing each pattern spectrum to 0/1 entries, using partial ordering with respect to size and occurrences to obtain a cumulative distribution (Torre et al., 2013), and then averaging over the surrogate realizations. The *p*-value of signature (*z*, *c*, *d*) is the corresponding entry of the *p*-value spectrum. Finally, the *p*-value spectrum is used in the Benjamini–Hochberg procedure to correct for multiple testing (Benjamini and Hochberg, 1995), where the number of tests is the number of occupied signatures (*z*, *c*, *d*) where (*z*, *c*+1, *d*) is not occupied.

The pattern counts found in the original data (Fig. 2, upper branch) are evaluated by assigning to each entry (signature) of the pattern spectrum the corresponding entry of the *p*-value spectrum (pattern spectrum filtering; PSF). See also section Consequences of spike count reduction on significance for the separation of significant and nonsignificant bins. We apply a significance level *α* = 0.05. If a pattern signature is assigned a *p*-value ≤ *α*, the corresponding patterns are considered potentially significant. These are further filtered by the pattern set reduction (PSR; Torre et al., 2013), which consists of conditional tests of each pattern given any other pattern surviving the PSF, to remove spurious FPs resulting as a by-product of the overlap of significant patterns and chance spikes. With this procedure, the number of significant patterns can be larger than the number of significant tests, because each significant signature may contain several patterns.

Finally, as a result of the explained steps, SPADE outputs significant STPs, together with their number of occurrences, the involved neurons, the lags between the pattern spikes, and the times of pattern occurrences.

When analyzing large-size experimental data, the FIM search for all possible patterns can result in obtaining millions, if not billions, of putative patterns (Porrmann et al., 2021). To reduce computation time, which is particularly relevant for large datasets, we require a minimum occurrence count *min_occ_* of each pattern size to be further considered after the frequent mining step. This parameter results as a rough estimate of the number of patterns expected by chance assuming independent stationary Poisson processes each with the average rate of each of the spike trains (Stella et al., 2019). Patterns with a lower number of occurrences are considered as spurious as they would be rejected anyway by the following statistical test. In addition, we fix the minimum number of occurrences of a pattern to be at least 10, i.e., 30% of the number of trials of the considered experimental data (see next section). The FIM output is then aggregated for the PSF and the pattern set reduction (Torre et al., 2013).

### Experimental data and preprocessing

**Figure 3.**
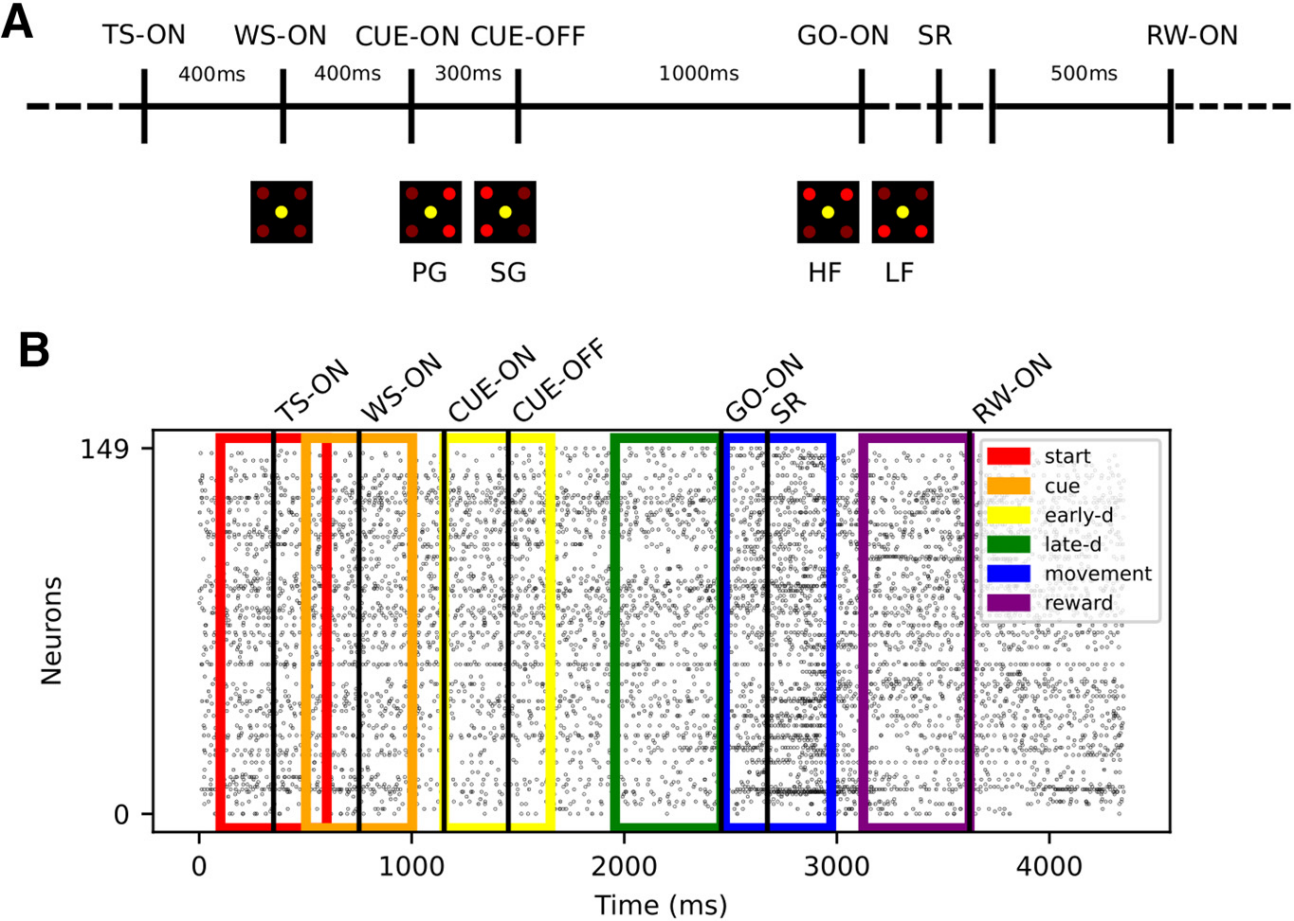
Experimental protocol and data preprocessing. ***A***, The trial start (TS) is self-initiated by the monkey. A waiting signal (WS-ON) prepares the monkey for the visual cue presented at CUE-ON, providing the grip type instruction (PG/SG). After 1000 ms, a second visual cue (GO-ON) is presented to the monkey, specifying the force needed to pull the object (HF or LF) and the GO signal. The switch release (SR) marks the beginning of the movement. The monkey touches the object and maintains the grip for 500 ms until the reward (RW-ON). The timing of the behavioral events SR and RW are variable and depend on reaction time and movement speed. ***B***, The panel shows the simultaneous spiking activity of all neurons (*y*-axis) over time (*x*-axis) for one example trial (first successful trial of session i140703-001) of trial type PGHF. Each dot indicates one spike. The trials are aligned to TS (TS-ON). The six colors rectangles represent the position of the six trial epochs (see legend).

We make use of experimental data in two respects: (1) we simulate data to test for FPs, and therefore extract statistical features of the experimental data to be included in the artificial data; (2) we analyze the experimental data for spike correlation by using the various surrogates. The experimental data were recorded during a reaching-and-grasping task from the pre-/motor cortex of two macaque monkeys, one female (monkey L) and one male (monkey N). Both monkeys were chronically implanted with a 100-electrode Utah array (Blackrock Microsystems). The experimental protocol is schematized in Figure 3*A* and was also published previously (Riehle et al., 2013; Brochier et al., 2018; Riehle et al., 2018). Monkeys N and L were trained to self-initiate a trial by pressing a start button (registered as trial start; TS). Then, after a fixed time of 400 ms, a visual signal (yellow LED) was shown, to attract the attention of the monkey (waiting signal; WS). After another 400-ms-long waiting period, a first visual cue (two LEDs on) was presented to the monkey for a period of 300 ms (from CUE-ON to CUE-OFF) indicating the grip type: full-hand side grip (SG) or two-fingers precision grip (PG). Followed by another waiting period of 1000 ms, the GO-signal was presented, containing also the information of the expected grip force (high, HF, or low, LF, by LEDs). The behavioral conditions were selected in a randomized fashion. The start of the movement of the monkey was recorded as the release of a switch (SR). Subsequently, the object touch (OT) and the beginning of the holding period (HS) are indicated. After 500 ms of holding the object in place, a reward (RW) was given to the monkey and the trial finished.

The experimental datasets considered here consist of two sessions (i140703-001 and l101210-001) of 15 min electrophysiological recordings containing around 35 trials per trial type, i.e., combinations of grip and force type. Each session is spike sorted using the Plexon Offline Spike Sorter (version 3.3). The spike data were extracted and are available on https://gin.g-node.org/INT/multielectrode_grasp.

We only consider neurons satisfying the following constraints: SNR > 2.5 (signal-to-noise ratio of spike shapes), average firing rate across trials <70 Hz. Hypersynchronous (artifact) spikes across electrodes arising at the sampling resolution are detected automatically, classified as artifacts, and removed as in Torre et al. (2016a). Only successful trials are retained. The two experimental sessions are analyzed separately. To perform a quasi time-resolved analysis, the trials are segmented into six 500 ms-long epochs that partly overlap and cover the entire trial. This procedure enables us to relate the data to the behavioral context (as in Torre et al., 2016a; and represented in Fig. 3*B*). Segments of the same epochs in the same trial type are concatenated and yield 24 (four trial types × six epochs) datasets per session, that are analyzed separately.

### Artificial nonstationary data

The simulated artificial datasets consist of nonstationary spike trains generated as two different point process models [Poisson process with dead-time (PPD); Deger et al., 2012, and as a Gamma process], each with similar single spike-train features as the experimental data but without precise time correlations. The particular point process models are chosen to account for the dead-time and regularity of the data. We generate as many spike trains as in the experimental data using the original firing rate profiles of the individual neurons. These are estimated with an optimized kernel density estimation as designed in Shinomoto (2010) and Shimazaki and Shinomoto (2010) on a single trial-by-trial basis.

Since the time-varying firing rates are an important property of experimental data which has to be considered in the “null-hypothesis”, we generate nonstationary spike data with the required ISI regularities. The PPD is a variation of the classical Poisson process wherein no spike is generated within an interval to the previous spike smaller than the dead-time *d*. The Gamma process has a shape parameter *γ* which is related to the intrinsic regularity/irregularity of the spike train (Nawrot et al., 2008). If *γ* > 1, the process is regular (i.e., CV < 1); if instead *γ* = 1, it coincides with the classical Poisson process with an exponential ISI distribution. We do not consider Gamma processes with *γ* < 1 (CV > 1), i.e., bursty spike trains because our experimental data did not contain such cases.

For the PPD data, we estimate the dead-time for each neuron and each combination of epoch and trial type separately by taking their minimum ISI. The nonstationary profile is obtained by the so-called “thinning” method. Specifically, the method first creates a PPD process of a rate equal to the temporal maximum and then consists in the rejection algorithm (of the single spikes) to simulate the continuous varying firing rate (Lewis and Shedler, 1979; Cardanobile and Rotter, 2010). For the data modeled as a Gamma process, we instead fix the shape factor for each neuron and each combination of epoch and trial type by estimating the CV of the process in operational time (Nawrot et al., 2008) and then transform the CV into the shape factor $\gamma =\frac{1}{{\text{CV}}^{2}}$, (van Vreeswijk, 2010). The process is generated in operational time and then transformed back into real time, thereby obtaining a nonstationary process. We then evaluate the regularity through the CV2 measure, which compensates for nonstationary firing rates (Holt et al., 1996). The resulting CV2 distribution of all neurons of the data, simulated as Gamma processes, is very close to the one of the experimental data (Fig. 8*A*, third inset). Note that the Gamma process does not have an absolute dead-time, however, for *γ* < 1, the process has a low probability of generating small ISIs and can be regarded as containing a relative dead-time (Nawrot et al., 2008). The resulting firing rates of the artificial data are, for both generative models, close to the ones of the experimental spike trains.

Each of the 24 datasets of the two experimental sessions (above, in Experimental data and preprocessing) is modeled using the two point process models PPD and Gamma, resulting in a total of 2 × 24 × 2 = 96 artificial datasets.

### Code accessibility

**Figure 4.**
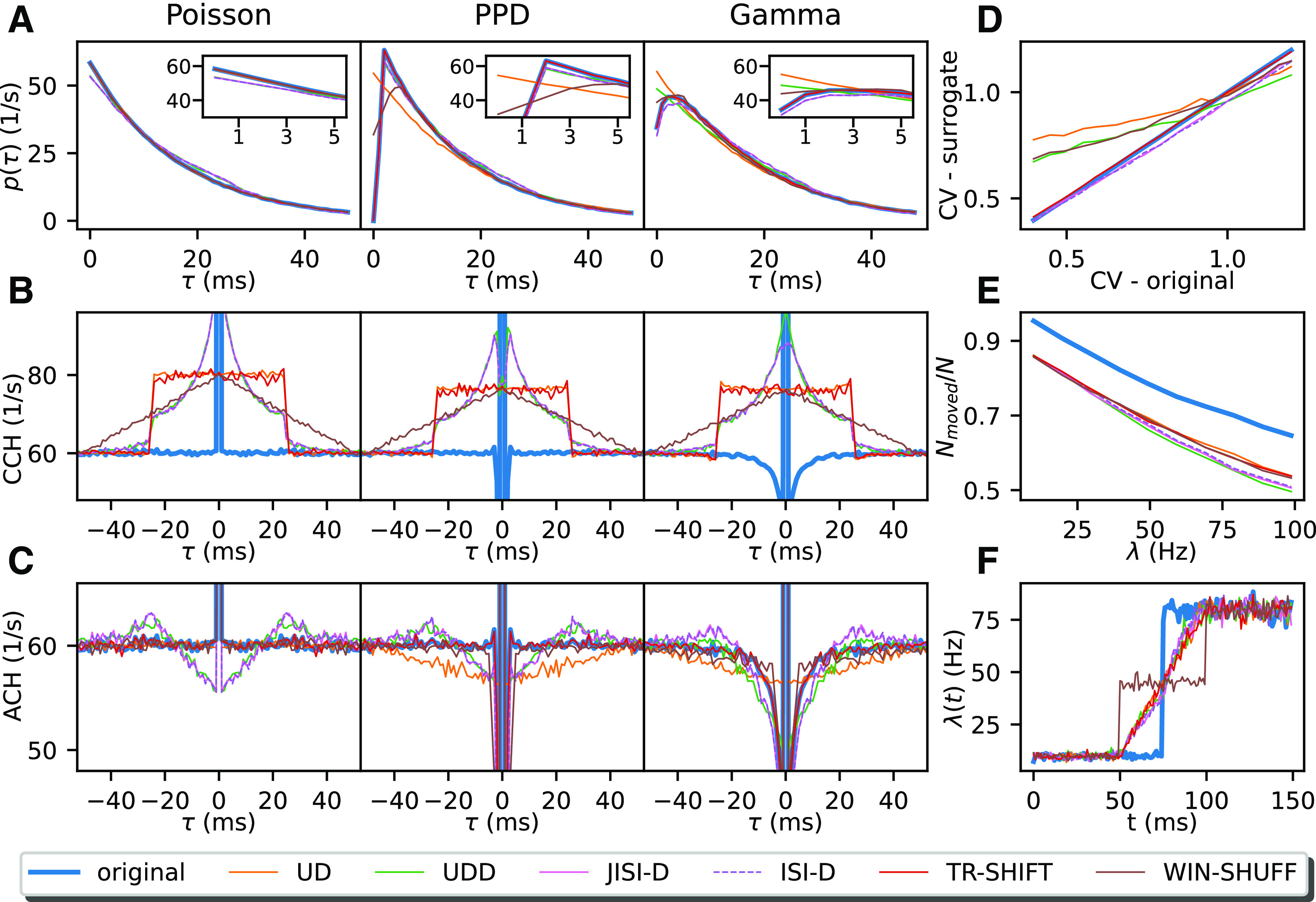
Overview of surrogate statistics. ***A***, The ISI distributions of original and surrogate spike trains (UD: orange, UDD: green, JISI-D: pink, ISI-D: violet, TR-SHIFT: red, WIN-SHUFF: brown) are shown as a function of the time lag *τ* in milliseconds (resolution of 1 ms). For each process, the corresponding spike trains have a firing rate of 60 Hz and an average spike count of 500,000 spikes. The ISI region smaller than 5 ms is shown in an inset at the upper right corner. ***B***, The panel shows the cross-correlation between the stationary spike train model (Poisson, PPD, and Gamma, in left, middle, and right column, respectively) with each of the surrogates (same color code as in ***A***), blue is the correlation with the original spike train with itself (i.e., the auto-correlation) as reference. ***C***, Auto-correlation histograms are shown before (solid blue) and after surrogate generation (colored lines). For ***B***, ***C***, the *x*-axis shows the time lag *τ* between the reference spikes and the surrogate spikes (***B***) and the other spikes in the spike train (***C***). For panels ***B***, ***C***, we use the same data as in panel ***A***. In panels ***D–F***, we only examine Gamma spike trains. ***D***, The panel displays the relation of the original CV (*x*-axis) against the CV of the surrogates (*y*-axis). Parameters are the same as in panels ***A–C*** (right), but we vary the CV ( $CV=1/\sqrt{\gamma }$) in steps of 0.05, ranging from 0.4 to 1.2 (*λ* = 60 Hz). ***E***, The ratio of moved spikes (*N*_moved_) over the spike count *N* is shown. We show it as a function of the firing rate from 10 to 100 Hz in steps of 10 Hz on the *x*-axis (*γ* = 1.23). ***F***, Conservation of the rate profile of a Gamma spike train (*γ* = 1.23) and its corresponding surrogates. The firing rate change is a step function, going from 10 to 80 Hz (10,000 realizations, spike train duration of 150 ms), and is computed as a PSTH with a bin size of 1 ms.

**Figure 5.**
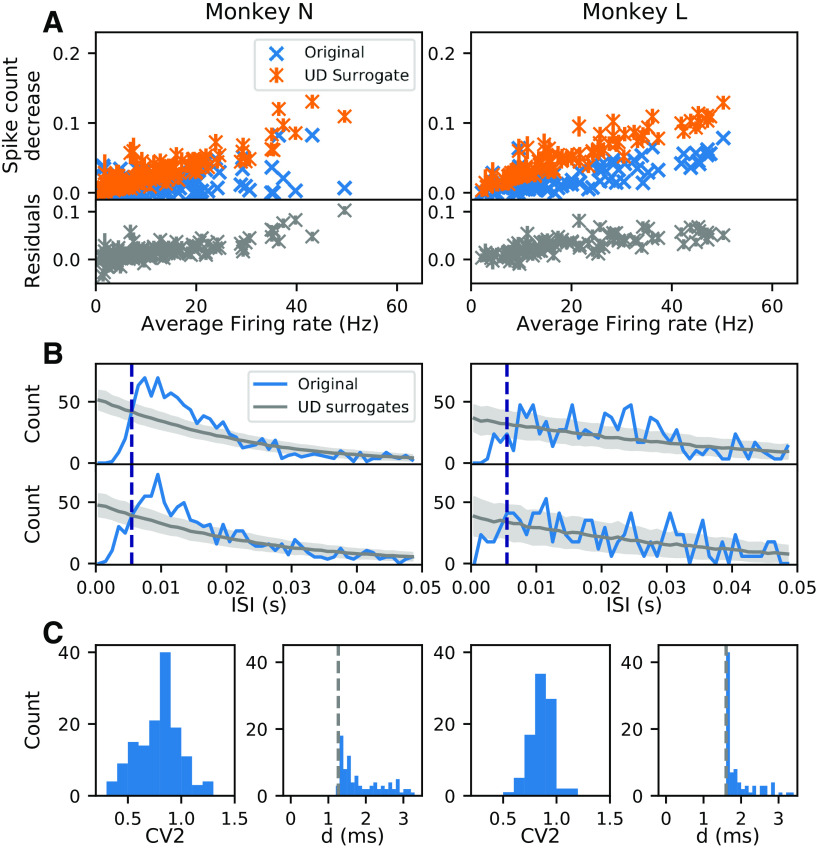
Modification of spike trains because of binarization. ***A***, Spike count reduction resulting from binarization and UD surrogate generation. Results from the analysis of two experimental datasets (sessions i140703-001 and l101210-001) in the movement epoch of the trial type PGHF of monkeys N (left) and L (right). Top panel, Spike count decrease as a function of the average firing rate. Blue crosses indicate the spike count reduction caused only by binarization of the original spike trains (one cross per neuron). Orange marks show the spike count reduction after surrogate generation by UD and binarization. The spike count reduction is normalized by the spike counts of the original continuous-time spike train. Orange bars indicate the SD of the spike count reduction calculated across 100 surrogates. Bottom panel: residuals (gray) computed as the spike count difference between the original binarized spike trains (blue) and the UD binarized surrogates (orange), normalized as in the top panel. ***B***, ***C***, Interval statistics of the data. ***B*** shows the ISI distribution of two neurons from monkey N (left) and two neurons of monkey L (right; in blue). Neurons represented are 7.1 and 16.1 for monkey N, 5.1 and 6.1 for monkey L, with channel-id.unit-id notation. In gray are the ISIs of the respective UD surrogate distributions with the mean (dark gray) and the SD of 500 surrogates in light gray. The bin size of the binning (here, 5 ms) is shown by the dashed dark blue line. In ***C***, the CV2 distributions are shown for all neurons in each of the datasets (***C***, left subpanel). ***C***, Right subpanels, Respective minimal ISI from the ISI distributions of all neurons. The dead-times assigned by the spike sorting algorithm are indicated by the dotted gray line (1.2 ms for monkey N and 1.6 ms for monkey L).

**Figure 6.**
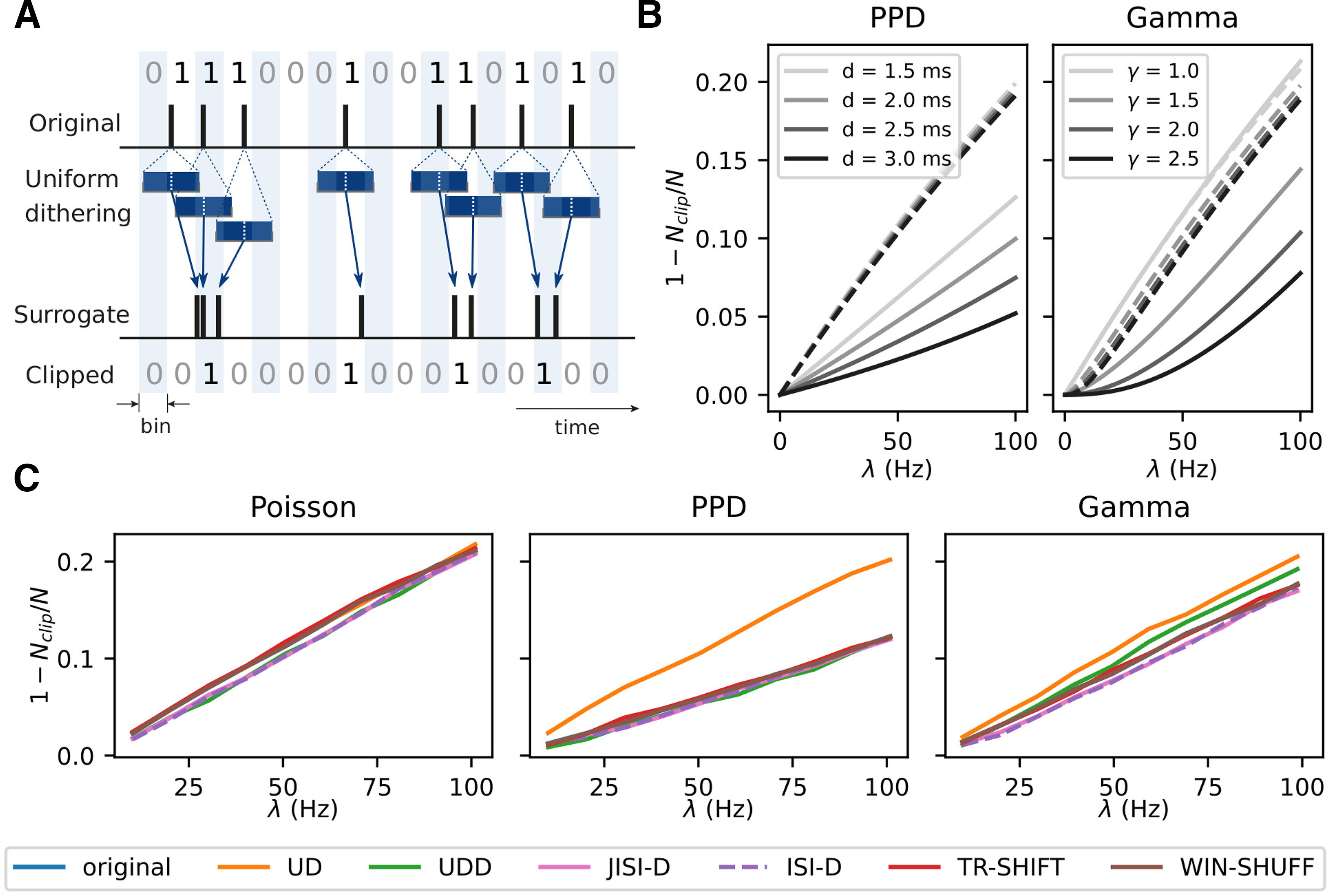
Origin of spike count reduction. ***A***, The sketch shows how a regular spike train is binarized. Below, illustration of how UD may change the spike times such that multiple spikes end up in single bins. The resulting binarized surrogate spike data are shown at the bottom. In contrast, because of the regular ISIs of the original process, its binned data are hardly losing spikes in comparison to the dithered version. ***B***, Analytical derivation of spike count reduction (after binning in 5-ms intervals and clipping) for renewal point process models (PPD, left and Gamma process, right; solid lines, respectively), each with four different parameter sets (PPD: d=1.5,2.0,2.5,3.0ms, Gamma: γ=1,1.5,2,2.5, different gray shades). The dashed lines show the same quantity for their UD surrogates. The firing rate of the processes is also varied and shown along the *x*-axis. The spike count reduction is shown on the *y*-axis, expressed as 1 – *N_clip_*/*N*, where *N_clip_* is the number of clipped spikes and *N* is the total number of spikes. ***C***, Spike count reduction of artificially generated spike train data of Figure 4 after binarization (bin width of 5 ms) together with the corresponding surrogates in different colors. The firing rate is constant for each spike train and varies across realizations from 10 to 100 Hz in steps of 10 Hz (along the *x*-axis). The spike train durations are fixed such that, given the firing rate, all spike trains have an expected spike count of 10,000 spikes.

**Figure 7.**
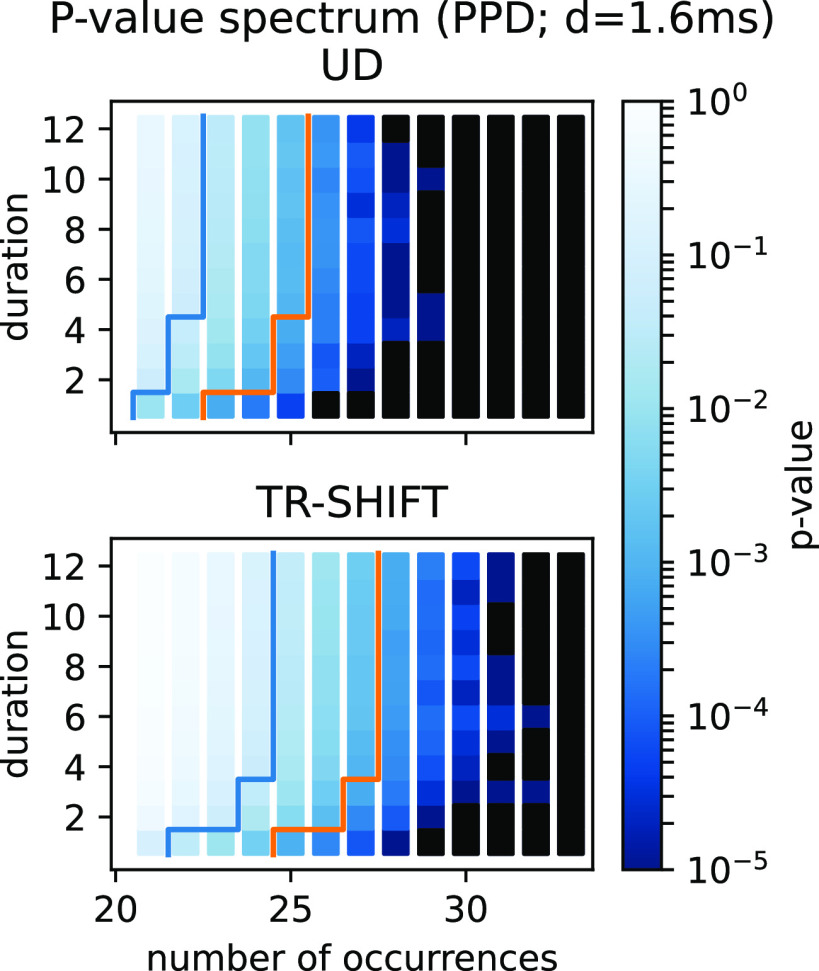
Consequences of spike count reduction on the *p*-value spectrum. The *p*-value spectra of stationary and independent PPD processes, using UD surrogates in the top panel and TR-SHIFT surrogates in the bottom panel. The *p*-value spectra are shown for mined patterns of size 3 only for a range of different pattern durations d (*y*-axis), and pattern counts (*x*-axis). The *p*-values are expressed by colors ranging from dark blue to light blue (see the color bar, identical for both spectra). The bin size is 5 ms. The results are from 20 realizations of *n *=* *20 parallel, independent, two seconds long PPD spike trains with parameters *λ* = 60 Hz, *d* = 1.6 ms. The *p*-value spectrum is derived from 5000 surrogates, dither parameter Δ = 25 ms. Further, we cut the spike train into trials of 100 ms for TR-SHIFT. The blue line indicates the limit of *α* = 0.05, to the right the *p*-values are below. The orange line shows the significance threshold after multiple-testing correction.

**Figure 8.**
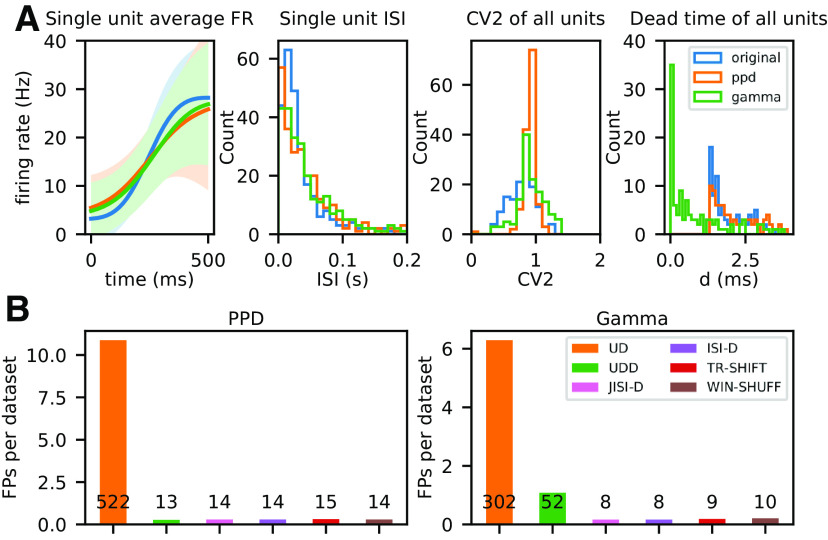
Evaluation and analysis of FPs across surrogate techniques for pattern detection with SPADE. ***A***, Comparison of statistics of the artificial data generated from experimental data to the experimental data. In blue, orange, and green we represent experimental, PPD, and Gamma data, respectively. Left graph, Average firing rate (PSTH smoothed) of a single unit (session: i140703-001, epoch: movement, trial type: PGHF, channel_id: 21, unit_id: 1) across one epoch of 500 ms across trials. Second from left, ISI distribution of the same single unit during the same epoch. Third from left, Average CV2 estimated trial wise for all neurons of the session. Fourth from left, Dead-time (minimum ISI) for all single neurons. ***B***, Numbers of FPs across surrogate techniques (on *x*-axis, color-coded) averaged over 48 ( 2 sessions×6 epochs×4 trial types) datasets, left for PPD and right for Gamma processes. The numbers above/inside the bars represent the total number of FPs found in 48 datasets, typically one FP pattern per dataset.

**Figure 9.**
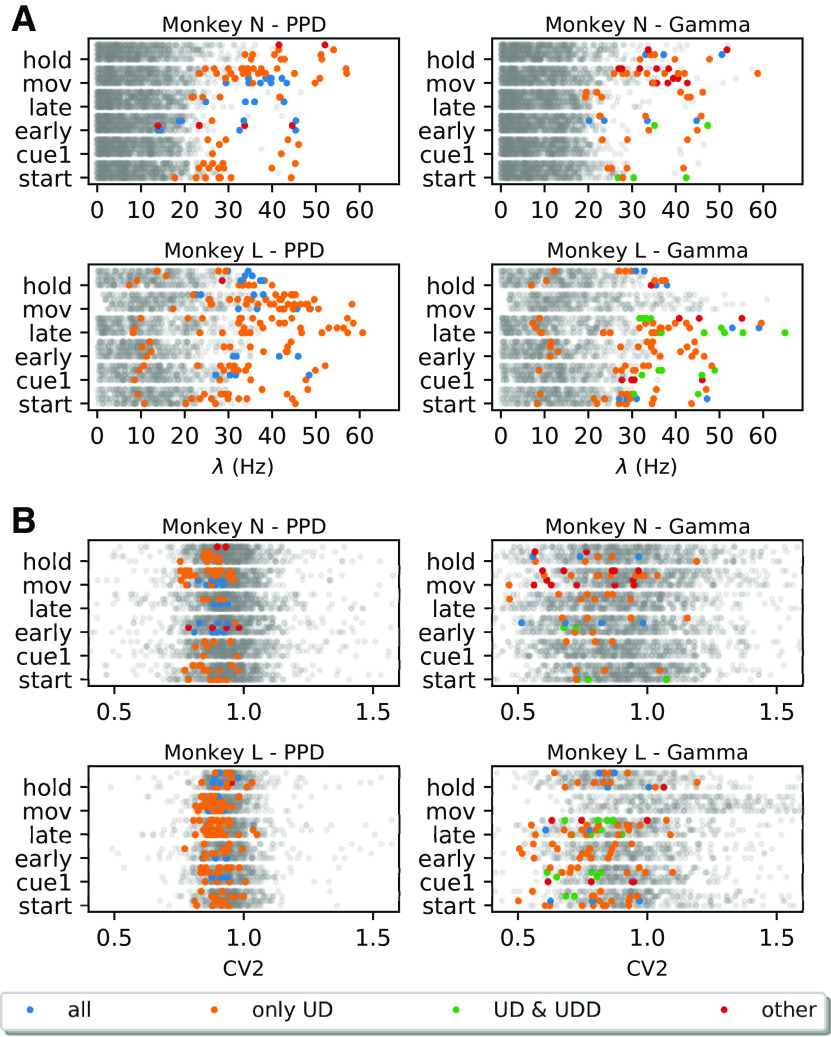
Average Firing rate and CV2 of neurons participating in FP patterns against all neurons. ***A***, Average firing rate of neurons for each monkey (N at the top and L at the bottom), epoch (*y*-axis), and behavioral type (for each epoch ordered as PGHF, PGLF, SGHF, SGLF). Left, PPD data. Right, Gamma data. Colored dots represent individual units involved in FPs: blue dots indicate the average firing rate of units involved in FP patterns found for all surrogate techniques, orange dots for UD surrogates, green dots for UD and UDD only, and red dots for other combinations of different surrogate techniques. Gray dots represent the average firing rate of individual units not involved in any FP. ***B***, Average CV2 of neurons for each monkey (N at the top and L at the bottom), same structure as ***A***.

**Figure 10.**
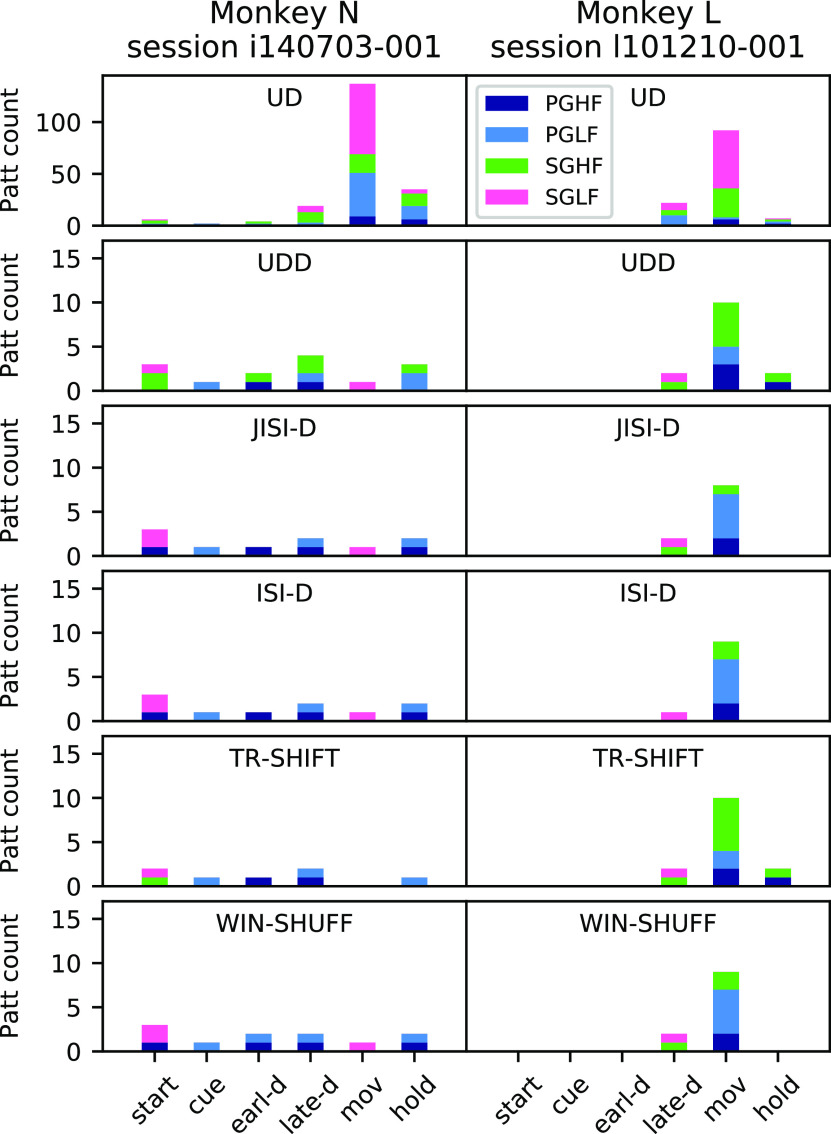
Analysis of experimental data. SPADE results for two sessions of experimental data: session i140703-001 (left) and session l101210-001 (right). Histograms represent the number of significant patterns detected by SPADE in each epoch (start, cue, early-delay, late-delay, movement, and hold), color-coded according to the grip type [precision/SG (PG/SG) and low/high force (LF/HF)]. Each row corresponds to one surrogate technique (note the different *y*-axis scale for UD).

The code to perform and reproduce the analyses presented in this study can be found at (https://github.com/INM-6/SPADE_surrogates), along with the code to reproduce the figures contained in this paper, i.e., Figures 3*B*, 4–10. Figures 1, 2, 3*A* are sketches created manually with vector graphics editors. The experimental data (analyzed in Results, Application to experimental data), can be found at https://gin.g-node.org/INT/multielectrode_grasp. The artificial data are generated from the experimental data within the SPADE_surrogates repository (Extended Data 1). The SPADE method and all implementations of the surrogate techniques are included in the Elephant Python package http://python-elephant.org. Regarding the computational cost, several improvements have been made for the performance of both, SPADE and the surrogate implementations (https://elephant.readthedocs.io/en/latest/release_notes.html; Porrmann et al., 2021). Nonetheless, depending on the size of the dataset and the number of surrogates employed, large analyses can still take up to several hours on a computer cluster.

10.1523/ENEURO.0505-21.2022.ed1 Extended Data 1 SPADE surrogates. Download Extended Data 1, ZIP file.

## Results

### Statistical comparison of surrogate methods

To get a better understanding of the effects of the surrogate methods on the statistical features of the spike trains, we first perform a comparison on stationary data. For this purpose, we simulate point process models with well-defined properties: a Poisson process as a reference, a PPD, and a Gamma process. The latter two are chosen to mimic the ISI distributions of the experimental data (Fig. 5*B*). Here, the processes are stationary to exclude further statistical aspects. We explore the effect of all six surrogate methods on the statistical properties of the stationary data (“original data”) for each neuron, each epoch, and trial type. The parameters of the data models are adjusted to be close to the experimental data and thus enable the discussion of the potential transfer to experimental data analyzed later (in Application to experimental data). Artificial data with nonstationary firing rates are analyzed in a later section (Performance of surrogate methods on nonstationary data).

Figure 4 summarizes the results on stationary, independent data. The columns refer to the different spike train models (Poisson, PPD, and Gamma, from left to right, respectively) and in Figure 4*A* the ISI distributions, in Figure 4*B* the cross-correlations between the artificial and the surrogate data, and in Figure 4*C* the auto-correlation of the artificial and the respective surrogate data for comparison. The fourth column displays a comparison of the CV of the artificial versus the surrogate data, the effectiveness of the displacement of the spikes through the surrogates, and the change of a rate step through the surrogate (from top to bottom).

#### ISI distribution

The ISI distributions (shown for *λ* = 60 Hz; Fig. 4*A*) indicate for all surrogate data an exponential decay, lower for short ISIs for JISI-D, ISI-D, and UDD. Moreover, for Poisson data, and in particular at high rates, the ISIs are often short. For the PPD process, UD dithers many spikes into the interval of up to 5 ms (see inset), corresponding to the bin width. The other surrogates, which preserve the dead-time, follow more closely the original ISI distribution. On the other hand, a Gamma process does not contain a strict dead-time but has a preferred ISI defined by the order of the process if *γ* > 1 (here *γ* = 1.23). The ISI distributions (Fig. 4*A*, right) are most changed for UD and UDD, whereas the other methods maintain the ISI distribution at small ISIs almost identical to the original one.

#### Similarity of artificial model and surrogate data

As a second step, we study the similarity for each of the three artificial datasets to their six surrogate datasets by generating cross-correlations between them. For the Poisson process (Fig. 4*B*, left), the different surrogates seem to generate again a Poisson-like process, since all surrogates move spikes around the original spike positions: uniformly within the dither window for UD or TR-SHIFT, or in a triangular fashion for WIN-SHUFF with a high probability around zero and an exponential-like decay for JISI-D, ISI-D, and UDD up to the dither window of 25 ms. In the case of JISI-D, ISI-D, and UDD, the dithering is limited by construction to the interval between the preceding and the following spike. Thus, for Poisson data, and in particular at high rates, the available dither window may not even be used completely, but only within the time interval between the two limiting spikes. Therefore, spikes are on average less moved and stay relatively close to their former positions (Fig. 4*E*).

For the case of PPD, the distribution of the spike shift from its original position (Fig. 4*B*, middle) is similar to the Poisson process case (left), but the probability that spikes stay close to their original position is slightly reduced (e.g., pink and dashed violet and green have a lower peak close to the center). Finally, for the Gamma process, spikes are shifted into short ISIs, but to a lesser degree for WIN-SHUFF. Thus, the shift of the surrogate spikes from the original data (Fig. 4*B*, right) is similar in Poisson and PPD models.

#### Auto-correlation

We examine the auto-correlations of the artificial processes and the versions modified by the surrogates to understand how ISI features are affected. For the Poisson process, the auto-correlations (Fig. 4*C*, left) for UD, TR-SHIFT, and WIN-SHUFF are flat except for the center peak, whereas JISI-D, ISI-D, and UDD show a decreased probability for very small ISIs, and then an increase up to the maximum dither width Δ = 25 ms with a peak above baseline. The reason for this difference is the limitation that spikes may not be exchanged in their order, as for the other methods. For example, if the reference spike is close to the following spike and further away from the preceding one, it will be more likely displaced backward in time than forward. This is also slightly visible in a difference of the ISI distributions (Fig. 4*A*, left), compared with the other methods. Moreover, the increase toward Δ above baseline is because of the shift of dither probability to higher time intervals. When looking at the PPD process, we notice instead that for JISI-D, ISI-D, and UDD, the auto-correlations show, as compared with the corresponding surrogates of the Poisson process, also reduced short ISIs, but to a lesser extent. UD moves spikes into small ISIs, but on a smaller scale than the Poisson process (Fig. 4*A*, left and middle, orange), and therefore shows a reduced probability when two spikes have a time difference of less than twice the dither parameter (here 50 ms). Finally, in the Gamma process, we have seen that the ISI distribution is maintained at small ISIs almost identical to the original one. Similarly, for the auto-correlations (Fig. 4*D*, right): TR-SHIFT is identical to the original process; JISI-D and ISI-D are mostly preserving the auto-correlations, but still have a small bump above baseline at around Δ because of the dither restriction not to dither beyond the former and the next spike. WIN-SHUFF has a sharp reduction of spikes after the reference spike, and UD has a dip around 0.

#### Coefficient of variation of ISIs

We learned that the ISI distributions are affected by most of the surrogates. Figure 4*D* illustrates how the CV of the surrogates differs in contrast to the original Gamma process (rate fixed to 60 Hz), where the CV ranges from 0.4 to 1.2 in steps of 0.05. Nonpreservation of the CV in the surrogate data as compared with the original data can be a potential source of FPs, in particular for very small CVs or CVs > 1 (Pipa et al., 2013). To facilitate the comparison, we also show the diagonal (blue). UD changes the CV the most, from the original 0.4–0.75, i.e., losing strongly its high regularity, and increases even more, with a low slope, to a maximum slightly over 1.0 for the original CV of 1.25, so here burstiness is reduced. WIN-SHUFF and UDD behave similarly to UD, but for CV = 0.4 of the original data, these surrogates have a lower CV than UD (Fig. 4*D*, orange, green, and brown lines above all others); moreover, UDD stays below UD for all CVs. WIN-SHUFF has a slightly higher slope and reaches a maximum still below the one of the Gamma process.

JISI-D, ISI-D, and TR-SHIFT start with identical CVs as Gamma, and TR-SHIFT keeps the same CV as the CV of the Gamma process for all CVs. However, JISI-D and ISI-D have a lower slope than the Gamma process, but still, reach high values ∼0.05 less than the highest CV at 1.25.

In summary, although the ISI distributions seem not to be strongly affected, the effect on the CVs can be very strong. For UD, UDD, and WIN-SHUFF, the CV slightly changes (in both directions), and for JISI-D and ISI-D, the CV decreases. A strong change in the CV of the surrogates can lead to FPs (Pipa et al., 2013). The CV is unchanged only for TR-SHIFT.

#### Ratio of moved spikes

Next, we study whether spikes are moved from their original bin in their surrogate realization. The reason for this interest is that if spikes are not sufficiently moved, correlations are not destroyed as intended, and thus may lead to false negatives. Therefore, we measure the ratio of the number of spikes that are displaced from their original bin position relative to the total number of spikes. We generate stationary data and its surrogate data and vary the firing rate (from 10 to 100 Hz in steps of 10 Hz; Fig. 4*E*). If two spikes exchange their bin positions, they are both considered as not moved. The spike ratio is also shown as a reference for two independent realizations of a Gamma spike train (*γ* = 1.23, blue line).

With increasing firing rates, the ratio of moved spikes decreases for the surrogates. Ideally, the surrogates should be similar to the effect attained on the original process, i.e., the colored lines in Figure 4*E* should be as close as possible to the blue line. However, none of the surrogate techniques meets this ideal setting, and there are constantly 10% fewer spikes moved as compared with the blue line, i.e., the ratio of moved spikes for two independent spike train realizations. Nonetheless, we observe for all surrogates that the ratio of moved spikes decreases with increasing firing rates, which corresponds to the fact that increasingly more bins are already occupied, and thus the resulting binned surrogate spike train is more similar to the binned original. UDD, ISI-D, and JISI-D displace fewer spikes, in particular for higher firing rates as compared with UD, TR-SHIFT, and WIN-SHUFF. The fewer the spikes that are not effectively displaced, the higher the peak at zero-delay of the cross-correlation of the stationary and the surrogate data (Fig. 4*B*). Almost 50% of JISI-D, ISI-D, and UDD are not moved at 100 Hz, and, for lower rates, they remain below the ratios of WIN-SHUFF, UD, and TR-SHIFT. As a consequence, we can expect that JISI-D, ISI-D, and UDD, in general, tend to yield more false negatives than WIN-SHUFF, UD, and TR-SHIFT.

#### Rate change in surrogates

Changes in the firing rate profile of the surrogates compared with the original data may be a source for FPs (Grün, 2009). An optimal surrogate method should follow as closely as possible the original firing rate profile. Therefore, we test here an extreme case where the original data have a rate step (as in Louis et al., 2010a), jumping from 10 to 80 Hz (Fig. 4*F*). We observe that for all surrogates but WIN-SHUFF the firing rate step is converted into a linear increase, which starts at 25 ms (dither parameter Δ) before the step and ends at 25 ms after the rate step. This behavior has already been derived analytically and observed in Louis et al. (2010a) for UD: it corresponds to the convolution of the original firing rate profile with the dither (boxcar) function. WIN-SHUFF introduces a second step in the firing rate profile as it generates a locally-stationary firing rate within the shuffling window (here 50 ms). We conclude that all surrogate techniques smooth the original firing rate profile, whereas WIN-SHUFF creates an additional intermediate rate step. Thus, for steep increases in the firing rate profiles, we have to expect the occurrence of FP patterns because of this smoothing.

#### Summary of the effects on the spike-train statistics of surrogates

We explored different aspects of the statistics of the surrogate spike data as compared with its original process. In general, surrogate data are not identical to the original data but change to a different degree. The effects for the three data models are summarized and not differentiated, since they are similar. These are listed in Table 1. Features that are preserved are indicated by “yes,” approximately preserved (“approx.”), and not preserved (“no”).

**Table 1:** Summary of the conservation of the statistical properties of the six surrogate techniques

Feature/method	UD	UDD	ISI-D	JISI-D	TR-SHIFT	WIN-SHUFF
ISI	No	No	Approx.	Approx.	Yes	Approx.
Dead-time	No	Yes	Yes	Yes	Yes	No
Auto-correlation	No	No	No	No	Yes	Approx.
Firing rate modulation	Approx.	Approx.	Approx.	Approx.	Approx.	Approx.
Spike train regularity (CV < 1; regular)	No	No	Approx.	Approx.	Yes	No
Spike train regularity (CV > 1; bursty)	No	No	Approx.	Approx.	Yes	Approx.

The degree of conservation is marked as yes/approx./no. The dead-time conservation is evaluated based only on the results of the PPD process, otherwise on the results for all data models (Poisson, PPD, Gamma).

### Impact on spike counts after spike train binarization

A typical step in the analysis of experimental spike data is to downsample them to the time-scale of relevance, e.g., millisecond resolution. This is typically done by binning the continuous spike trains to bins of a few milliseconds width, resulting in spike counts per bin. In further analysis steps, these data often have to be reduced to 0–1 sequences (e.g., as described for the SPADE method in Materials and Methods), thus the bin contents are reduced to 1 if one or more spikes are in a bin (“clipping”), or to 0 for no spike. Thus, we now explore whether and how the binarization step affects surrogate data. For doing so, we compare the spike counts per neuron before and after the binarization step, for both the experimental and the surrogate data. We notice that for some neurons the total spike counts of the UD surrogates are much lower than those of the original data. Further analysis of this aspect shows that the higher the firing rate of a neuron the larger the spike count reduction and thus the corresponding mismatch. Figure 5*A* shows that for two different datasets, each from a different monkey, we find a spike count mismatch of up to 10% between the UD surrogates and the original data (Fig. 5*A*, bottom, gray). Such a difference in the spike count is troublesome, since it is expected to lead to a reduced pattern count in the surrogates as compared with the original data and, thus, is expected to yield an overestimation of the significance of patterns.

#### Origin of spike count reduction

The UD procedure as such does not delete any spikes, only the binarization step does. In this regard, the latter step is crucial: when applied to the original and the surrogate data, it leads to different spike counts. Here, we aim to understand why this is the case. One potential reason is the change of the ISI statistics of the spike trains with and without dithering, as already analyzed for stationary spike models described above, (in ISI distribution). Figure 5*B*, shows the ISI distribution for two example neurons of the experimental data (in blue; right for monkey N, left for monkey L) and for comparison, the ISI distributions of the uniform dithered surrogates (gray). In the experimental data, the ISI distribution is peaked at a certain ISI, here between 5 and 10 ms, but the in ISI distributions of the surrogate data are decaying exponentially, and thus also fill small ISIs. This indicates the fact that the original spike trains are more regular than the surrogates and so small ISIs have a lower probability. The regularity of the experimental data is confirmed by the measurements of the CV2. Indeed, the CV2 distribution of all neurons of both two datasets (Fig. 5*C*, left subpanels, in both columns) is rather below 1, i.e., more regular than Poisson.

In addition, the distributions of the minimal ISI of each neuron per dataset (Fig. 5*C*, right subpanels of the two columns) exhibit a minimal ISI of 1.3 ms for monkey N and 1.6 ms for monkey L. This corresponds to the dead-times of the spike sorting algorithm that cannot resolve overlapping spikes. The different dead-times for the two monkeys are because of a different number of sample data points considered for spike sorting (Brochier et al., 2018). The corresponding ISI distributions of the surrogate data (Fig. 5*B*) show that there are ISIs smaller than the minimum ISI of the respective experimental data. Thus, the dithering procedure generates shorter ISIs in the UD surrogates than in the original data.

To verify our interpretation that the combination of UD and binarization causes the spike count reduction, we perform a similar analysis on artificial data, i.e., simulated PPD and Gamma spike trains. For simplicity, we now choose both of a constant rate but adapt the dead-times or shape factors, respectively, to account for the ISI features of the experimental data.

Figure 6*C* shows the spike count reduction (expressed as 1 – *N_clip_*/*N*, where *N_clip_* is the number of clipped spikes and *N* the total number of spikes) that results after binning (5 ms) of the PPD (left) and Gamma process (right) with (dashed) and without (solid) application of UD. The respective analytical derivation can be found in Stella et al. (2021). The graphs show the spike count reduction as a function of the firing rate of the processes. The PPD model shows a higher spike loss the higher the firing rate, and a lower spike reduction for larger dead-times. The uniformly dithered PPD show for all dead-times an increase in the spike reduction with higher firing rates, but to a larger extent than for the original PPD processes. The Gamma process (right) for *γ* > 1 shows a similar result as for the PPD: an increase of spike count reduction for increasing firing rates, and the larger the shape factor, the lower the spike count reduction. The increase is rather parabolical compared with the PPD. The Poisson process (light gray, *γ* = 1) shows a much larger and linear increase of spike reduction with rate, more strongly than for Gamma processes with *γ* > 1 (darker grays).

Thus, (1) why does a Poisson-like process lose more spikes through binarization than a process with a non exponential ISI distribution, and (2) why does UD lead to a loss of spikes compared with the original experimental data? As shown above (Fig. 5*B*), UD generates a more Poisson-like ISI distribution. Such processes contain spikes that follow each other in short intervals, which in turn would lead to more than one spike in a bin. The following clipping then reduces the spikes to 1 (for illustration, see Fig. 6*A*). A PPD process has a strict dead-time which prevents such small intervals between the spikes, thus fewer spikes are discarded in the binarization procedure. The closer the duration of the dead-time to the bin size, the more unlikely is that two spikes are dithered into the same bin, and the loss of spikes is reduced.

#### Spike count reduction in different spike train models

Figure 6*C* illustrates the spike count reduction for all types of surrogates applied to the three different spike train models described above: the higher the rate the more spikes are lost. However, there are some differences in the degree of spike loss for different data models, which we now discuss separately.

For Poisson data, the spike count loss increases approximately linearly with the firing rate for all surrogates. This happens as well for the Poisson process (blue), up to 20% for a rate of 100 Hz. Only JISI-D, ISI-D, and UDD overall have a slightly lower loss. In the case of the PPD process, the loss of spikes (Fig. 6*C*, middle) is considerably reduced as compared with the other models. This is true also for its surrogates, besides UD (orange), which loses more spikes. This may be explained by the ISI distributions that we observed in Figure 4*A* UD dithers many spikes into the interval of up to 5 ms (see inset), corresponding to the bin width. For the Gamma process, the spike loss for the different surrogates (Fig. 6*C*, right) is higher than for PPD but lower than for Poisson. UD loses the most, JISI-D and ISI-D the least. One method (WIN-SHUFF) preserves the spike count from the original spike train by construction. For TR-SHIFT, the differences in spike count are negligible.

As UD surrogates evidence a strong spike count reduction in the context of binarization, we expect UD to yield a large number of FPs in the SPADE PSF test. Further, UDD surrogates might lead to FPs in the case of regular data that do not exhibit a dead-time (e.g., Gamma spike trains). The study of the similarity of the surrogates to the original processes shows that JISI-D, ISI-D, and UDD might lead to fewer patterns detected, i.e., an underestimation of significance. Moreover, we expect FPs for WIN-SHUFF surrogates when the original firing rate profiles have a steep rate increase. The technique preserving the most statistics without showing any disadvantages is TR-SHIFT.

#### Consequences of spike count reduction on significance

Above we have shown that by UD we get a loss of spike counts because of the binarization. Now we show how this spike loss leads to a reduced expected number of spike patterns and thus to FPs. For doing that we create 20 times the *p*-value spectrum for a dataset containing 20 independent, stationary PPD spike trains and average over the obtained *p*-value spectra, once derived by UD (Fig. 7, top panel) and also by TR-SHIFT (Fig. 7, bottom panel). Figure 7 shows a cut through the 3d-*p*-value spectrum for the mined patterns of size 3, for different pattern occurrences (*x*-axis), and different durations (*y*-axis). The *p*-value spectra show that the *p*-values change from high values for low number of occurrences to low values for higher number of occurrences (from left to right, color-coded accorded to the color-bar on the right). The blue lines indicate in each of them the isoline of the significance threshold of *α* = 5*%*, and the threshold corrected for multiple testing in orange. The blue staircase-like line is always to the left of the orange staircase-like line approximately with a difference of three occurrences. We observe that the two lines are far more to the right for TR-SHIFT (approximately by two occurrences) than for UD. Thus, for UD fewer pattern occurrences are needed to classify a pattern as significant than for TR-SHIFT, e.g., for a duration of *d *=* *4, 25 occurrences are needed for UD (top) in contrast to 28 for TR-SHIFT (bottom). Or in other words, if the surrogates lead to a loss of spikes as for UD, the number of patterns required to become significant is lower. This also may lead to an increase of FPs through UD. We also did this analysis for the other four surrogate methods and could observe that they all behave similarly to TR-SHIFT (not shown here).

From the analyses performed here, we conclude that UD is not an appropriate surrogate method for spike data that either contain a hard dead-time or have a regular spiking behavior, as motor cortex data tend to have (Mochizuki et al., 2016). Therefore, we now deepen our evaluation of surrogate methods described above by analyzing the impact of these on the SPADE analysis.

However, before we move on, a note of caution concerning the use of the terms “false positives” and “false negatives” may be necessary: in the experimental data we do not have an independent “ground truth” telling us which patterns are the “real” ones. Thus, in principle, one cannot speak of false positives or false negatives, one can only speak about underestimation or overestimation of significance probabilities. However, we still use the term “false positive” to be compatible with the experimental literature we are relating to. In the artificial data, we can only generate data from a null-hypothesis, where patterns occur just by chance. Therefore, any patterns found to be significant in these data are considered false positives, and there can be no false negatives.

### Performance of surrogate methods on nonstationary data

Next, we apply the six surrogate techniques to artificial datasets that are generated based on two experimental datasets (Brochier et al., 2018) to study the effect of the surrogate methods on the occurrence of FPs in the case of nonstationary data (Louis et al., 2010a). The experimental data are two sets of recordings from ∼100 parallel spike trains from macaque monkey motor and premotor cortex during performance of a reach-to-grasp behavior (Riehle et al., 2013), explained in Materials and Methods, Experimental data and preprocessing. In this analysis, we account for firing rate changes by generating artificial spike trains with the same firing rate profile of the experimental data (thus nonstationary processes). The generated data thus reproduce from the experimental data both single neuron and population response profiles. In addition, we use two nonstationary point processes which model ISI, dead-time, and firing regularities of the experimental data.

Thus, we simulate nonstationary artificial data with the same firing rate profiles as the experimental data, and use as point process models (1) the PPD to mimic the dead-time of the data (because of spike sorting), and also (2) Gamma processes to account for their CVs. Before we show the results for the SPADE analysis of these artificial independent data, we show for a dataset from a movement epoch (PGHF) where the most firing rate changes are observed, the similarity of the artificial data to the experimental data. Figure 8*A* summarizes the features such as firing rate modulation, ISI, CV2, and dead-time for the experimental (blue), the PPD (orange), and the Gamma process (green). Clearly, the artificial data are not completely identical to the original data, but very close. The firing rate modulation (left panel) is relatively well kept, as well as the single unit ISI (second panel from left), albeit the PPD and the Gamma data also include small ISIs. The CVs of the data are relatively close in the mean, although the experimental data still have a broader distribution with lower values (third panel from left). The right panel shows the distributions of the smallest intervals. The original and the PPD are very similar with a clear dead-time of 1.6 ms, whereas for the Gamma process, spikes occur also close to 0 ms after another spike. These small differences also need to be considered when comparing the SPADE analysis results from the artificial data (Fig. 8*B*) and the experimental data (Fig. 10).

#### FP analysis on artificial nonstationary data

The artificial data analyzed here for FPs are generated independently and hence all observed spike patterns occur by chance and are considered as FPs. The SPADE analysis is performed on all datasets, and separately with each of the six surrogate techniques. We set the bin size to 5 ms, the maximum pattern duration to 60 ms, the significance level to *α* = 0.05, and use 5000 surrogates. For all surrogate techniques, we set the dither parameter to Δ = 25 ms.

Figure 8*B* shows the number of patterns retrieved for the PPD (left) and the Gamma datasets (right). For each point process model, we show the total number of FPs of all 96 datasets for each of the six surrogate methods. Results show that we retrieve for all surrogate techniques but UD a small number of FPs. For UD, we get for the PPD process in Figure 8*B*, left: UD (522) and for Gamma: UD (302). For the rest of the surrogates, we get for PPD around 14 FPs and for Gamma around 9, besides UDD (52). The latter was expected from our observations described above, (in Spike count reduction in different spike train models). Thus, concluding from these results, we expect in the analysis of the experimental data to get a similar level of FPs as shown here.

For further analyses, given these observations, we form four groups of FP types depending on which surrogate techniques they are expressed in. The first and predominant group are the FPs present only in the SPADE analysis performed with UD surrogates, represented in orange in Figures 8*A* and 9. Second, we group FPs present in all surrogate techniques (in blue). Third, in the case of Gamma data, we distinguish a subset of FPs found with both UD and UDD surrogates (in green). Finally, we pool all FPs present in any other combination of surrogate analysis (in red). To get an understanding of the rate properties of neurons that contribute to the FPs, we consider their average firing rates (over time and trials) and the group that they belong to (Fig. 9*A*). In general, we find FPs in all analyzed datasets, but four (monkey L, movement, Gamma, all conditions). We observe that almost all neurons involved in FPs have an average firing rate higher than 20 Hz. Neurons belonging to the first group (UD only) are the largest set and are present for both monkeys, both data models, and almost all datasets. The second group (all) is present for both monkeys and models but is larger for PPD. The third group (UD and UDD) is present for both monkeys only for the Gamma model. This was already expected, given the higher spike count reduction, for UD and UDD in the case of Gamma spike trains (see section Spike count reduction in different spike train models). We also inspect the CV2, averaged over trials, of units involved in FPs (Fig. 9*A*). FPs occur in neurons with a relatively low CV2 (0.7 < CV2 < 1), but this is not the case for neurons with very low CV2s (CV2 < 0.7; especially for monkey N). Neurons with CV2 > 1 are (almost) never involved in FP patterns.

In summary, we observe that the surrogate technique leading to most FPs is UD, followed by UDD (only in the case of Gamma data). Neurons exhibiting an average firing rate higher than 20 Hz, and having a CV2 < 1 are predominantly involved in FPs. Moreover, there is a small amount of FPs detected using all other surrogate techniques, which is expected given a certain significance threshold. Nonetheless, regular and high-rate neurons are more prone to raise the FP rate (Harrison and Geman, 2009).

### Application to experimental data

As the last step, we apply SPADE with the six surrogate techniques to the two sessions of experimental data introduced in section Materials and Methods, Experimental data and preprocessing. Here, our goal is to analyze with SPADE experimental data, for which we do not know the ground truth (i.e., the presence and amount of significant patterns) and show the differences resulting from the application of the surrogate techniques in the significance testing. In Figure 10, we present the found number of significant patterns for each epoch and trial type (different colors). The results are shown for each monkey separately since their data differ in terms of CV2, dead-time, and firing rates (Figs. 8, 9). The number of patterns across all datasets (24) per monkey is UD (N:203, L:121), UDD (N:14, L:14), JISI-D (N:10, L:10), ISI-D (N:10, L:10), TR-SHIFT (N:7, L:14), WIN-SHUFF (N:11, L:11). Note, for comparison to Figure 8, one needs to add the numbers of the two monkeys. Thus, we detect more patterns (almost double the amount) in the analysis of experimental data than in the nonstationary artificial data, except for UD (and UDD on Gamma data).

A first observation is that the amount of significant patterns found using UD is much higher (note different *y*-axis scale) for both monkeys as compared with the other five surrogate techniques. Patterns occur mostly during the movement (mov) epoch where the firing rates are highest. Thus, given the calibration results from the former section, a large amount of those are likely FPs.

Taking from now on into consideration all surrogate techniques but UD, for monkey N (Fig. 10, left column) we find patterns across all epochs, almost for all surrogates. The pattern numbers are relatively similar within and across epochs. Very specific are the pattern occurrences during the movement epoch (Fig. 10, left column, pink). In fact, the same pattern occurs for SGLF behavioral context in all surrogates, but TR-SHIFT. During the start epoch, all surrogates show patterns in relation to SGLF, but some (UDD and TR-SHIFT) are also in relation to SGHF, and others (JISI-D, ISI-D, and WIN-SHUFF) in relation to PGHF. In the cue epoch, all surrogates find patterns in relation to PGLF trials (Fig. 10, left column, light blue). During early delay (earl-d) all surrogate techniques find patterns for PGHF trials, in addition, for UDD, a pattern for SGHF, and one in PGLF trials for WIN-SHUFF. During the late waiting epoch (late-d) patterns occur only in PGHF and PGLF trials (Fig. 10, left column, blue and light blue), but we also observe patterns in SGHF trials for UDD (Fig. 10, left column, green, second row). Finally, during hold, we find patterns in PGLF trials for all surrogate techniques. In addition, we find a pattern in SGHF trials for UDD and a pattern in PGHF trials for JISI-D, ISI-D, and WIN-SHUFF.

For monkey L (Fig. 10, right column) most patterns occur during the movement epoch for PGHF, PGLF, and SGHF, however in slightly different combinations. During the late phase of the waiting period (late-d) four out of the five surrogates find the same patterns (one for SGLF and one for SGHF). During the hold epoch only for UDD and TR-SHIFT, we find the same patterns, one for PGHF and one for PGLF. We do not detect any significant patterns in the start, cue, and early delay epochs.

Interestingly, for both monkeys, the significant patterns are specific to the epochs, i.e., identical patterns do not repeat in different epochs, but the patterns are different in the temporal delays of the spikes and are mostly composed of different neurons. Previous studies on this experiment (Riehle et al., 2018; Fig. 2) showed that monkey L has on average a shorter reaction time than monkey N, and has shorter and more pronounced rate increases within the movement epoch. In contrast, monkey N shows patterns already in the start epoch, and the number of detected patterns remains almost constant throughout epochs.

## Discussion

The generation and use of surrogate data has become an important methodological approach to data analysis when the data are complex and there are no reasonable simple hypotheses that can be used for the statistical evaluation. This holds in particular for complex experimental data, i.e., massively parallel recordings during behavior and which do not contain hundreds of trials because the monkeys do participate in the experiment only for a limited time.

Of course, it is still possible to use comparably simple spike-train distributions as null-hypotheses to test for significant repetitions of spatiotemporal spike patterns, which will find more significant patterns with much less effort (Staude et al., 2010; Russo and Durstewitz, 2017). However, there may be several potential reasons for a significant deviation from such a simple null-hypothesis, in particular when it assumes stationarity and does not include the obvious effects of co-variation of neural firing rates with the experimental condition or stimulus presentation (Grün, 2009; Pipa et al., 2013; Elsayed and Cunningham, 2017).

Generative statistical models for more complex null-hypotheses have occasionally been introduced in the literature, for example, to test the significance of experimental findings that are presented as evidence for a more complex, but still rate-based “population coding,” but may be explained just by the first and second moments of the vector of neural firing rates (Elsayed and Cunningham, 2017). Here, we are interested in spatiotemporal spike patterns that cannot be explained by the rates at all, including potential correlations of all orders. For this purpose, the most reasonable procedures for generating sufficiently complex null-hypotheses probably have to be based on surrogate methods, in particular temporal dithering or jittering.

In this study, we perform a comparison of six surrogate techniques (Fig. 1) which are used in the analysis of parallel spike trains: uniform dithering (UD), uniform dithering with dead-time (UDD), window shuffling (WIN-SHUFF) both newly introduced, joint inter-spike interval dithering (JISI-D; Gerstein, 2004), inter-spike interval dithering (modified from Gerstein, 2004), and trial shifting (Pipa et al., 2008; Harrison and Geman, 2009). Such surrogate approaches have the goal of destroying the exact spike timing relations between neurons. We quantified which statistical features of the experimental spike trains are conserved in surrogates, by examining the ISI distribution, the auto-correlation, the cross-correlation, the firing rate modulations, the ratio of moved spikes, and the coefficient of variation (Table 1). These were evaluated on stationary artificial data (Poisson, PPD, and Gamma spike trains) that contained relevant features of the experimental data (dead-time and regularity; Fig. 4). Additionally, we observed that UD does not preserve the spike count when the spike trains are binarized and which leads to a very strong spike count reduction for the PPD model, not for the Poisson model, and less for the Gamma model.

The issue of spike count reduction shown for UD on stationary, independent data is particularly relevant, as it might influence the statistical test result. Looking more in detail into real experimental data, we have shown that the usage of UD as a surrogate technique, followed by binarization (binning and clipping) of the spike train, leads to a mismatch in the spike counts between the experimental and the surrogate data (Fig. 5). The spike count reduction is worthy of attention and increases with the firing rate, which we verified analytically and through simulations (Figs. 4*A*, 6*B*). Moreover, we showed that two factors play a major role in the spike count reduction: the neuronal dead-time and the CV (Fig. 6*B*).

Evaluation of spatiotemporal patterns is a tricky problem and has been discussed controversially in the literature (Oram et al., 1999; Baker and Lemon, 2000; Segev, 2003; Kumar et al., 2010; Quaglio et al., 2018). When considering the problem in the context of the statistical evaluation of spatiotemporal spike patterns using SPADE, we observed that the spike reduction in the UD surrogates in combination with binarization of the spike data leads to fewer pattern occurrences in the surrogate data compared with the original data, which in turn may lead to an overestimation of pattern significance in the original data (Fig. 7). The ultimate consequence of this problem is the occurrence of FPs. Fortunately, SPADE is a modular method: different types of surrogates can be used, while the mining algorithm and the testing steps stay identical. For this reason, we were able to analyze the same datasets by using different surrogates, to compare the results.

Thus, using SPADE, we tested all six surrogate techniques for FPs in nonstationary data. These were based on two experimental sessions of the motor cortex of macaque monkeys involved in a reaching-and-grasping task (Riehle et al., 2013; Brochier et al., 2018). We stress the importance of generating test data that are very similar to experimental data, to closely model all features that typically lead to complications when trying to implement the null-hypothesis of conditional independence given the firing rates (Grün, 2009). The features that play a key role in false significance estimation are the statistical properties of the single-neuron spike train and of the population. In particular, we account for firing rate increases or decreases by generating nonstationary spike trains with the same firing rate profiles as the experimental data. This allows us to preserve both single-neuron firing properties and also common gain modulations or firing rate correlations across the entire population, which are are ubiquitous properties of experimental datasets (Ecker et al., 2014; Rabinowitz et al., 2015; Mochizuki et al., 2016; Riehle et al., 2018). Moreover, the nonstationary point processes we propose in this study account for the single neuron statistics, such as ISI, dead-time, and firing regularities, which are also fundamental properties of experimental spike trains (Nawrot et al., 2008; Cardanobile and Rotter, 2010; Deger et al., 2012).

These realistic, but artificial, data serve to identify the strengths and weaknesses of the tested surrogate techniques. In this case, we modeled both experimental sessions as PPD and Gamma processes, with firing rate modulations, dead-times, and regularities estimated for each neuron from the experimental data (Fig. 8*A*). The analysis of these data with SPADE led to a large number of FPs when employing UD. However, all other surrogate techniques showed a considerably low number of FPs. A minimal number of FPs is to be expected, as it is inherent to any statistical test. Thus, we conclude that UD is not appropriate for its employment within the context of the SPADE analysis, whereas all other surrogate techniques can be considered valid.

Finally, we analyzed experimental data from Brochier et al. (2018). UD in this context leads to a large number of detected patterns (Fig. 10). Given the results obtained from the previous sections, analytically and through simulations, we consider these patterns as putative FPs (taking into consideration that in the case of experimental data we have no ground truth at hand). In contrast, employing the other surrogates the number of patterns detected is much smaller than using UD. Still, the number of patterns is larger than for the analysis of the corresponding nonstationary artificial data with these surrogates. Given also our previous results, we consider the patterns detected in the experimental data by the alternative surrogate techniques as significant, i.e., the patterns do not result from any overestimation of the significance. This is confirmed by the fact that the patterns, retrieved for UDD, JISI-D, ISI-D, WIN-SHUFF, and TR-SHIFT, show almost identical participating neurons, lags, and occurrence numbers. Hence, we conclude that the different surrogates, although they move the spikes in different ways, lead to an almost identical significance level.

We conclude that UDD, JISI-D, ISI-D, WIN-SHUFF, and TR-SHIFT are appropriate for the detection of spatiotemporal spike patterns in the SPADE analysis, and UD is not. Furthermore, we suggest TR-SHIFT as the surrogate method of choice for the SPADE analysis, because it is a technique that (1) is easy to explain and to implement; (2) reflects more closely the hypothesis of temporal coding; (3) reproduces exactly the most relevant statistical features of a spike train (Table 1; Fig. 4); (4) is as conservative as the other methods that we propose; and (5) employs fewer parameters than the other techniques with the same performance.

Of course, the employment of surrogates is not only restricted to the context of a SPADE analysis but was used in other studies for the evaluation of correlations (Gerstein et al., 1978; Hatsopoulos et al., 2003; Pipa and Grün, 2003; Pipa et al., 2003, 2007; Pazienti et al., 2007, 2008; Maldonado et al., 2008; Smith and Kohn, 2008; Grün, 2009; Harrison and Geman, 2009; Louis et al., 2010a; Dann et al., 2016; Torre et al., 2016b). Still, the choice of a particular surrogate technique has to be done appropriately and cautiously case by case. Not only because the statistical test might produce FPs (or FNs), but also because the concrete null-hypothesis distribution represents the model that is to be falsified. The degree of how conservative or liberal the statistical analysis can be, through the choice of the surrogate technique, becomes then not only a feature of the test but more a scientific question per se regarding neural coding.

Several studies have already investigated the impact of different surrogate techniques in the context of spike time correlations. For example, in Louis et al. (2010b), the authors evaluated the influence of surrogate techniques on cross-correlation analysis of two parallel spike trains; in Grün (2009) and Louis et al. (2010a), the focus was on the effect of surrogate techniques on synchronous events in the context of the Unitary Events analysis (Grün et al., 2002a,b; Pipa and Grün, 2003; Pipa et al., 2003, 2007, 2013). Because of the results of these studies, we have concentrated on surrogate techniques that preserve the firing rate profile of the experimental neurons. Methods such as spike train randomization (within single trials; Grün et al., 2003), spike exchange (across neurons or trials; Harrison et al., 2007; Smith and Kohn, 2008), ISI shuffling (within and across trials; Nádasdy et al., 1999; Masuda and Aihara, 2003; Ikegaya et al., 2004; Rivlin-Etzion et al., 2006), spike shuffling across neurons (within-trial; Nádasdy et al., 1999; Ikegaya et al., 2004) do not fulfill our requirements (Grün, 2009). Other methods are designed to preserve the auto-correlation of a spike train, with the assumption of stationarity and the Markov property of a process (Ricci et al., 2019; Perinelli et al., 2020).

Some studies have already shown evidence of problems arising from the application of UD, such as the nonpreservation of the ISI distribution (Louis et al., 2010a), in particular in the case of the Poisson process (Platkiewicz et al., 2017), but not in the context of multiple parallel spike trains, or in the context of binarization. We extended previous similar comparative studies of surrogate techniques (done only for pairwise correlations; Louis et al., 2010a) to the context of precisely timed higher-order spike correlations. In considering repeating patterns with time delays between spikes across several neurons, it has been argued that the processing of information may be reflected in the presence of delayed higher-order correlations in parallel spike trains, in particular, in the context of the synfire chain model (Abeles, 1991; Bienenstock, 1995; Diesmann et al., 1999; Izhikevich, 2006; Oettl et al., 2020). Given our present state of ignorance concerning the detailed computational functioning of the brain, the use of statistical methods like SPADE seems to be almost the only way to find out whether the detailed, precisely timed coordination across several individual neurons is important for information processing beyond the more global temporal covariations of neural activity that appear between cortical layers and across cortical areas.

We have started to apply SPADE to a large set of experimental data to investigate the presence of spatiotemporal patterns and evaluate their relation to behavior. The first encouraging results are shown in Figure 10.

## Synthesis

Reviewing Editor: Nicholas J. Priebe, University of Texas at Austin Decisions are customarily a result of the Reviewing Editor and the peer reviewers coming together and discussing their recommendations until a consensus is reached. When revisions are invited, a fact-based synthesis statement explaining their decision and outlining what is needed to prepare a revision will be listed below. The following reviewer(s) agreed to reveal their identity: Robbe Goris.

The authors have responded to the reviewers’ comments, incorporating substantial changes to their analysis and interpretations that have increased the clarity of this study. The authors present a strong case that generating null hypothesis distributions based on the uniform dithering technique leads to a higher rate of false positive spike patterns than generating distributions based on other techniques (such as trial shifting), when spike trains are analyzed with the SPADE method.
